# Direct visualization of dispersed lipid bicontinuous cubic phases by cryo-electron tomography

**DOI:** 10.1038/ncomms9915

**Published:** 2015-11-17

**Authors:** Davide Demurtas, Paul Guichard, Isabelle Martiel, Raffaele Mezzenga, Cécile Hébert, Laurent Sagalowicz

**Affiliations:** 1 Interdisciplinary Centre for Electron Microscopy, Swiss Federal Institute of Technology (EPFL), Lausanne 1015, Switzerland; 2 Swiss Institute for Experimental Cancer Research (ISREC), School of Life Sciences, Swiss Federal Institute of Technology (EPFL), Lausanne 1015, Switzerland; 3 Department of Health Science and Technology, ETH Zurich, Zurich 8092, Switzerland; 4 Nestlé Research Center, Vers-Chez-Les-Blanc, Lausanne 1000, Switzerland

## Abstract

Bulk and dispersed cubic liquid crystalline phases (cubosomes), present in the body and in living cell membranes, are believed to play an essential role in biological phenomena. Moreover, their biocompatibility is attractive for nutrient or drug delivery system applications. Here the three-dimensional organization of dispersed cubic lipid self-assembled phases is fully revealed by cryo-electron tomography and compared with simulated structures. It is demonstrated that the interior is constituted of a perfect bicontinuous cubic phase, while the outside shows interlamellar attachments, which represent a transition state between the liquid crystalline interior phase and the outside vesicular structure. Therefore, compositional gradients within cubosomes are inferred, with a lipid bilayer separating at least one water channel set from the external aqueous phase. This is crucial to understand and enhance controlled release of target molecules and calls for a revision of postulated transport mechanisms from cubosomes to the aqueous phase.

## Abstract (web summary)

Dispersed lipid self-assembly can form various types of particles, including cubosomes, which are useful for drug delivery. Here, Demurtas *et al*. visualize their three-dimensional structure, showing two continuous water channels separated by lipid bilayers and the mechanism of particle stabilization.

## Graphical abstract

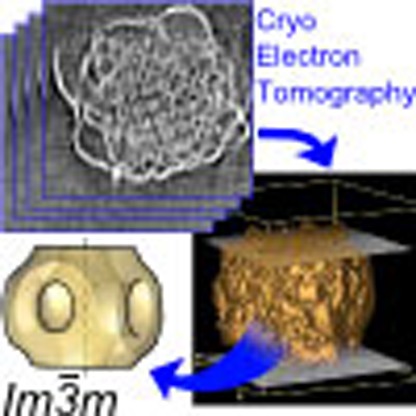

Amphiphilic lipids can self-assemble into a variety of structures^1^,^2^,^3^. In particular, the inverse bicontinuous cubic phases and their applications and role in nature have received significant attention^4^,^5^,^6^,^7^. These structures have been reported to occur spontaneously in mitochondrial membranes, in stressed or virally infected cells^5^,^8^ and are believed to be essential for understanding vital mechanisms such as cell fusion^6^,^9^ and food digestion^3^,^10^. They are used for the growth of protein crystals to study their structure^11^. In medicine and industrial applications, they can control the release of drugs and flavours^12^, and have been shown to enhance the yield of the Maillard reaction products^4^. There is overwhelming evidence that the macroscopic properties of lipid structures depend on their fine structure. It is therefore of prime interest to develop methods for the accurate determination of those structures.

The most commonly used technique for this purpose is small-angle X-ray scattering (SAXS). This method relies on constructive interferences, in the reciprocal space, from a large number of ordered scattering planes, and therefore does not provide a straightforward visualization of the structure in the direct space.

Further limitations arise when materials are dispersed into water to form submicrometer particles. The scattered signal is then often restricted due to the small size of the objects, which leads to a low coherence and limited information^13^.

In standard cryo-transmission electron microscopy (cryo-TEM), the signal results from the complete thickness of the vitrified sample, which limits the resolution and subsequent structural interpretations.

To circumvent these limitations, we used cryo-electron tomography (CET) to unveil the three-dimensional (3D) organization at nanometric scale of self-assembled structures formed by a dispersed phase composed of biologically and industrially relevant unsaturated monoglycerides. CET enables the reconstruction of 3D information in the native state and the investigation of large structures with unique topologies. In colloidal science and in biology, the development of CET gives access to the fine structure of biological features such as organelles at a high resolution of only a few nanometres^14^,^15^,^16^,^17^,^18^.

Emulsification of monoglyceride/surfactant mixtures in water results in the formation of particles with an interior displaying liquid crystalline organization. We performed CET on those particles to demonstrate the presence of internal bicontinuous cubic structure and to study the water–particle interface. Employing a subtomogram averaging on the liquid crystalline region, we directly characterized the internal 3D organization and compared it with the prevailing mathematical model of the bicontinous cubic structure demonstrating that the particle interior is constituted by two continuous water channels separated by lipid bilayers. We then investigated the interface organization between dispersed particles and the water involved in particle stabilization, which has a strong influence on the release of active elements solubilized within the cubosomes. It was found that the transition between the particle structured core and outer vesicles is made possible by the presence of interlamellar attachments (ILAs). This work enables to unambiguously determine the structure of the particles interior and forges a new understanding of the structural gradient within the particles.

## Results

### Cryo-electron microscopy of self-assembled structures

**Figure 1:**
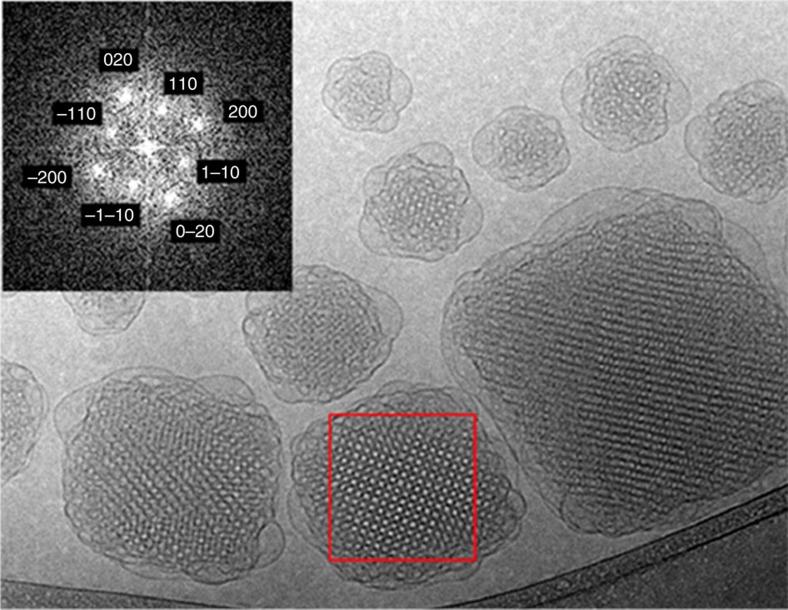
Representative cryo-TEM image of the cubosme dispersion used in this study. Note the presence of a well-ordered structure (for example, red box) in the particle inside and of a vesicular structure close to the interface with the water matrix. The inset shows the fast Fourier transform (FFT) of the red box area and it is used for the structure determination of the liquid crystalline particles, independently confirmed by SAXS analysis. Scale bar, 100 nm.

In view of applying CET to cubosomes, it is crucial to select compositions and dispersion conditions leading to well-ordered structures and to a lattice parameter as large as possible. We used an optimum amount of polyglycerol ester to tune these parameters (Supplementary Figs 1 and 2). Cryo-TEM images reveal that particles are internally ordered and have a diameter ranging from 100 to 500 nm (Fig. 1). They coexist with vesicles and attached vesicular structures (Fig. 1; Supplementary Fig. 3), as usually reported^13^,^19^. As indicated by SAXS and cryo-TEM crystallographic analyses, the internally ordered particles have a space group symmetry and a lattice parameter of about 16 nm (Fig. 1; Supplementary Figs 1 and 4).

In previous literature, the self-assembled structures of lipid mesophases and in particular their bicontinous nature have been inferred from crystallographic arguments based on X-ray scattering, although not unequivocally. The independence of the two distinct water channel networks in cubic phases has been demonstrated before, but indirectly via the introduction of transport membrane proteins at the lipid bilayers^12^. Stimuli-triggered opening of those protein pores linked the two independent water channel networks, which resulted in faster diffusion of ions and molecules. No direct observation of the water and lipid network of the bicontinuous phases has yet been produced.

### Bicontinuous cubic structure visualized by CET

Here we show how it has been possible to directly visualize the 3D networks by CET and we demonstrate unambiguously the presence of bicontinuous cubic structure and the presence of two independent water channels.

**Figure 2:**
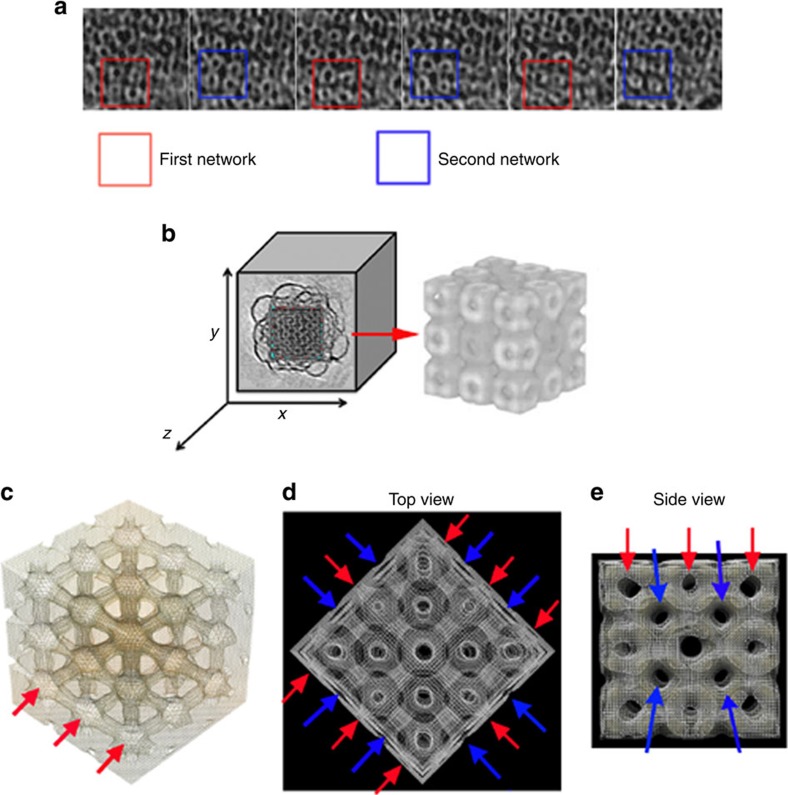
3D organization of the liquid crystal region inside a cubosome. (**a**) Sequence of images extracted from the tomogram along the *z* direction. It shows both the first (red box) and second (blue box) network in alternate position indicating the sequence of the channels (we adopted arbitrarily the term of ‘first and second' to differentiate the two interdependent networks). (**b**) Central core of the original tomogram used for the subtomogram averaging process and its 3D reconstruction showing the unit cells. (**c**) Extract of the tomogram showing one of the two water channel network. Red arrows show the nodes and the enlargement of the water network. (**d**–**e**) Top and side view of the filtered 3D reconstruction where the pores belonging to the first (red arrows) and second network (blue arrows) are indicated (rendering of the subtomogram averaging in Supplementary Movie 1).

Cubosomes in a size range of 100–300 nm were chosen for 3D reconstruction, since their low thickness leads to a high signal-to-noise ratio. The sequences of images extracted from the tomogram in the *z* direction show holes belonging to the water channels (Fig. 2a). When progressing along the *z* direction, the periodic network is shifted by half of the diagonal of the cube (*a*√3/2, where *a* is the lattice parameter). This provides a direct and separate visualization of the two independent networks indicated by the red and blue box (first and second network).

To reconstruct and visualize the native 3D structure of the crystalline part of the cubosome, image-processing techniques were used to increase the contrast and the resolution of this region. It was first determined that the liquid crystal has a periodicity of 16.8 nm (Supplementary Fig. 5) for the particular particle studied in Fig. 2. Then, the central region of tomogram volume was selected to extract 150 boxes. Finally, we took advantage of the structure periodicity and symmetry to duplicate and rotate each box accordingly, which compensated for missing information in the axial orientation^20^. An average map was created from all extracted boxes, as presented in Fig. 2b–e. This subtomogram averaging approach in direct space on a single cubosome differs fundamentally from the indirect space summation by photon interferences, which underpins scattering methods.

The resulting sub-tomogram averaging reveals unprecedented detail the 3D organization of the bicontinuous structure. The ‘top' and ‘side' view of the filtered 3D image clearly show how the channels are organized in two interdependent networks, indicated by the red and blue arrows (Fig. 2d,e). Moreover, one of the water channels is represented in Fig. 2c, giving insight on localization and diffusion of potential solubilized hydrophilic molecules.

### Comparison between the mathematical and the CET reconstruction

The bilayer of bicontinuous cubic phase forms infinite periodic minimal surface structures that can adopt the gyroid (G), diamond (D) and primitive (P) surfaces^21^. We used here the concept of periodic nodal surface, which for the inverted bicontinuous cubic structures leads to a structure very close to the corresponding periodic minimal surface^22^. For the P surface, the equation of the periodic nodal surface is^23^:


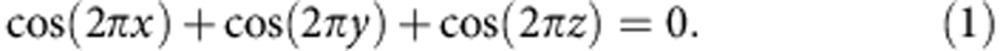


**Figure 3:**
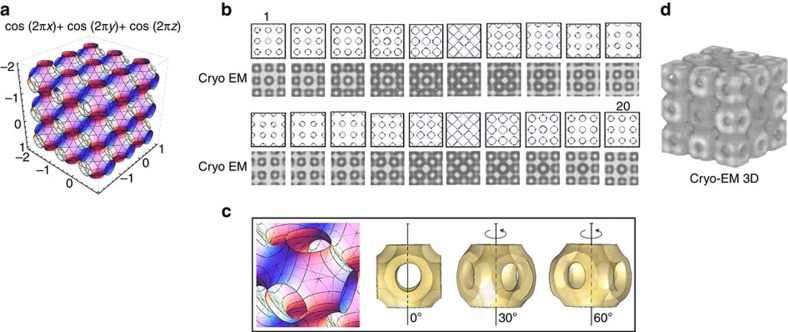
Comparison between mathematical model and cryo-ET 3D reconstruction. (**a**) Minimal surface type of cubic structure. (**b**) Comparison between the decomposition series of the model and the stack series obtained by CET of the liquid crystal region. (**c**) Mathematical model of the unit cell and their 3D reconstruction projections obtained by CET. (**d**) 3D reconstruction by CET.

Figure 3a gives the 3D image of the P surface obtained using equation (1).

A direct comparison in 3D between the average map obtained by electron tomography (Fig. 3d) and the 3D theoretical surface (Fig. 3a) is difficult and cannot be quantitative. Therefore, following an approach taken from condensed material sciences, we took slices from the experimental tomogram and decomposed in a series of 20 images showing the progression along the *z* direction, describing one unit cell. These images can be compared with the curves generated by equation (1) at different fixed *z* values (Fig. 3b). The similarity between the series is excellent, thus demonstrating that the bicontinuous cubic structure really corresponds to the primitive surface.

With the CET coupled with the subtomogram averaging, it has been possible to visualize the 3D structure of the unit cell and to compare it directly with the mathematical model. This confirms the bicontinuous character of the phase and its similarity with the P type.

### Interface between cubosomes and water

Having resolved the interior structure of a dispersed cubic structure, we focused on the structure of the interface. Numerous reports have been devoted to solving the interfacial structure between a cubic phase particle and water. A first stabilization mechanism was proposed by Larsson^21^ and was described in more details later on by Anderson and Jacobs^24^. Gustaffson *et al*.^19^ suggested that the Anderson model (Supplementary Fig. 6) could be seen as an idealized model for cubosome stabilization, but could not explain why the surface of cubosomes is consistently observed to be crowded with disordered vesicular structures (Fig. 1). Later on, based on freeze fracture images, Angelov *et al*.^25^ proposed another model, which again did not involve disordered vesicular structures but particles were built with repeated elementary structures also present at the surface. In a later study, Boyd *et al*.^26^, using scanning electron microscopy, deduced that the surface was compatible with the model proposed by Angelov *et al*. In the present work, CET makes it further possible to directly visualize the organization of the surface between the cubic phase particle and the surrounding water, and to study how lipids build structural order and complexity when going from the water/particle interface to the interior.

**Figure 4:**
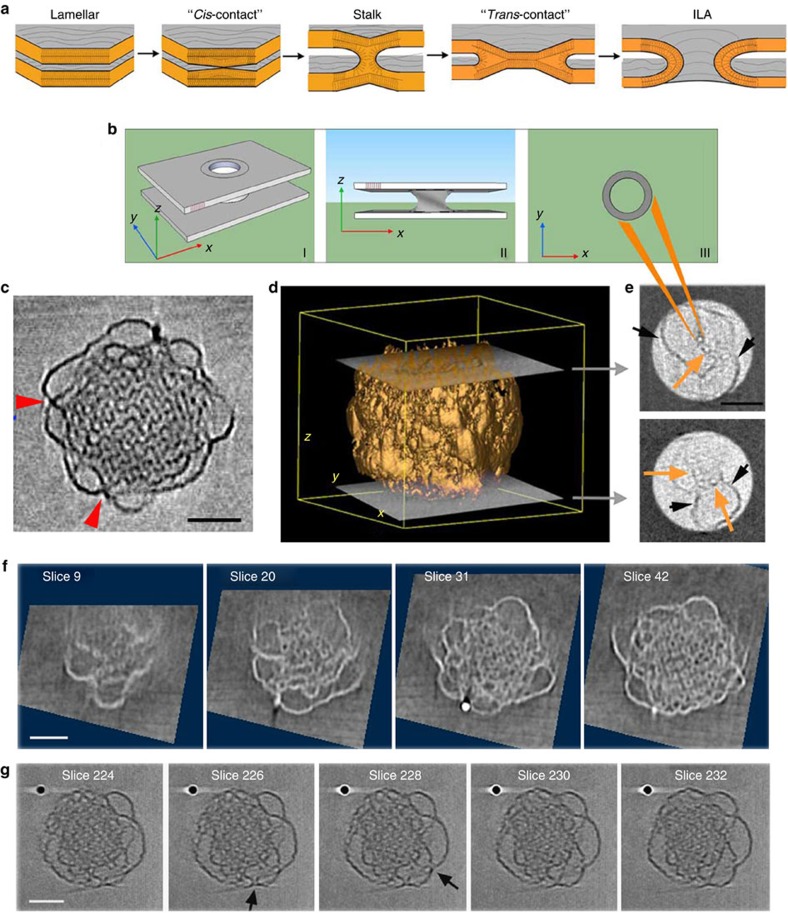
Membrane fusion initiates layer formation of bicontinuous cubic phase. (**a**) Schematic of the membrane fusion process. (**b**) Model of the ILA (I), side (II) and top views (III). (**c**) *Z*-slice extracted from the ‘central' part of the tomogram showing membrane fusion events (red arrows). (**d**) 3D rendering of the whole volume of the cubosome with the position of the two top and bottom extreme slices of the shell (**e**), where ILAs (orange arrows) and the curved lamellar layers (black arrows) are visible. (**f**) The sequences extracted from the tomogram shows at the beginning only few ILAs close to the interface (slice 9). In the successive slices, the number of ILAs increases presenting an irregular organization (slice 20). At the centre of the particle, the packing of the ILA in the deep layers forms the bicontinuous cubic structure in which the water channels are well organized (slice 42). Slices were cut normal to the ILA axis. (**g**) Sequences of slices extracted from a tomogram of a small cubosome where visible ILAs are oriented at 90° (black arrows) from the ones of Fig. 4e,f. All scale bars, 50 nm.

We propose a new model for cubosome stabilization that CET enables us to validate. In the spatial transition from the external shell to the ordered core of the cubosome, we were able to identify structures that are similar to the transient intermediates reported in the fusion of lamellar membranes^27^. In cubosomes, structural transitions from lamellar to reverse bicontinuous cubic phase (Fig. 4a), involving stalks and interlamellar attachments (ILAs) as intermediates, have been so far reported only as a function of time^27^. ILAs were also reported to be present during the formation of sponge and discontinuous cubic phases (Q_L_)^28^ or in bulk phase^29^. An initial ‘cis' (apposed) membrane contact between two bilayers first occurs, followed by the formation of a stalk^30^. By contact of the ‘trans' (non-opposed) monolayers of the original two bilayers in the stalk, a transmembrane contact is formed (Fig. 4a). A pore or ILA^9^ appears by rupture of the transmembrane contact. As the number of ILAs per unit area increases when moving towards the particle centre, they organize in squares lattices, yielding the final bicontinuous cubic phase without any open bilayer^29^.

Figure 4c shows an experimental tomogram slice passing through the core of the particle. It is clear that the structure gradually evolves from disordered vesicular-like structures on the outer surface to a highly ordered structure in the core. The simulated model of an ILA (Fig. 4b) indicates that ILAs should appear as circular holes when viewed from the top (Fig. 4bIII). Membrane undulations and fusion can be observed in the centre slice (Fig. 4c), although the visualization of ILAs has been difficult due both to the low resolution and the missing wedge effect^31^. The thickness of the vitreous layer is a parameter that contributes to the low contrast of the CET. For this reason, small particles or intermediate structures are more favourable for a direct visualization of the membrane fusion (Supplementary Fig. 7; Supplementary Movie 2). The volume rendering (Fig. 4d) shows that the structure of the object is elongated, preventing the visualization of details in the external shell of the cubosome. Only in the top and bottom slices of the tomogram (Fig. 4e), which are tangent to the cubosome surface (Fig. 4d), ILAs could be clearly visualized as small circles (orange arrows). Lipid bilayers, which are not parallel to the slicing plane, appear as thick black lines (black arrows).

Animations of the electron tomographic reconstruction indicate that the number and order of the ILAs increase when moving from the particle top (or bottom) to the centre (Fig. 4f; Supplementary Movie 3) in agreement with the proposed model.

In Fig. 4c, membrane fusion events are observed but due to the missing wedge, the same ILAs cannot be observed on the side view and ILAs are observed from bottom (top view, Fig. 4e). In Fig. 4g, the small thickness of the object allows to see ILAs observed along the side view confirming their presence.

Therefore, our results demonstrate that the stabilization of cubosomes involves the transition between lamellar liquid crystalline and cubic phases generated by membrane fusion of the lamellar structure and ILAs.

## Discussion

In literature, cryo-TEM experiments performed on cubosomes obtained with different stabilizers^13^,^32^, different processes of manufacturing^33^,^34^, different lipids (phytantriol and phospholipids)^35^,^36^ and different bicontinuous space groups^13^ demonstrate the presence of disordered vesicular structure at the interface (Supplementary Figs 8–11). Supplementary Fig. 12 shows membrane fusion events for a dispersion for which the particles have a diamond structure (space group). This dispersion was heated for 20 min at 120 °C to obtain a more stable dispersion^37^. Similar features are also observed for phospholipid dispersions^36^.

The model proposed by Anderson (Supplementary Fig. 6) can be seen as a particular case of the stabilization mechanism described in this study and as the simplest means to stabilize cubosomes. In particular, vesicular caps prevent from having the lipophilic part of the emulsifier in contact with water at the water cubosome interface and a regular repartition of ILAs, at the interface, insures the transition to the bicontinuous cubic structure. Supplementary Fig. 6, and the similarity between the schematic and experimental image, strongly suggests that the Anderson model applies when no apparent lamellar structure is present. We can therefore conclude that the mechanism of stabilization proposed here (including the Andersson model) applies for different compositions and processes and is probably very general for lipids. For non-lipids, the situation may be completely different. In particular, in cubosomes obtained using block-copolymers, no lamellar structure is observed and both water channels are open to the outside^38^. However, the physical/chemical rules for block-copolymer cubosomes are very different since those are stable after removing water, which is not the case for lipid cubosomes.

For lipids, the reason dictating the presence or absence of a large lamellar structure at the interface is not fully clear. It could be that since the diamond bicontinuous cubic structure (symmetry) is stabilized using more lipophilic lipid, less vesicular structures could be present compared with the primitive one (). However, as discussed before, large amount of lamellar structure at the interface are also observed for the structure (Supplementary Fig. 12 and images in de Campo *et al*.^39^). The nature of the stabilizer is probably more important than the space group. Proteins seem to provide less vesicular structures (Supplementary Fig. 9), while partially hydrolyzed lecithin increases it (Supplementary Fig. 8). Likely, proteins interact less with monoglyceride (or phytantriol) than partially hydrolyzed lecithin. In addition, it was reported that the amount of vesicular structure at the interface increases with Pluronic amount^19^.

**Figure 5:**
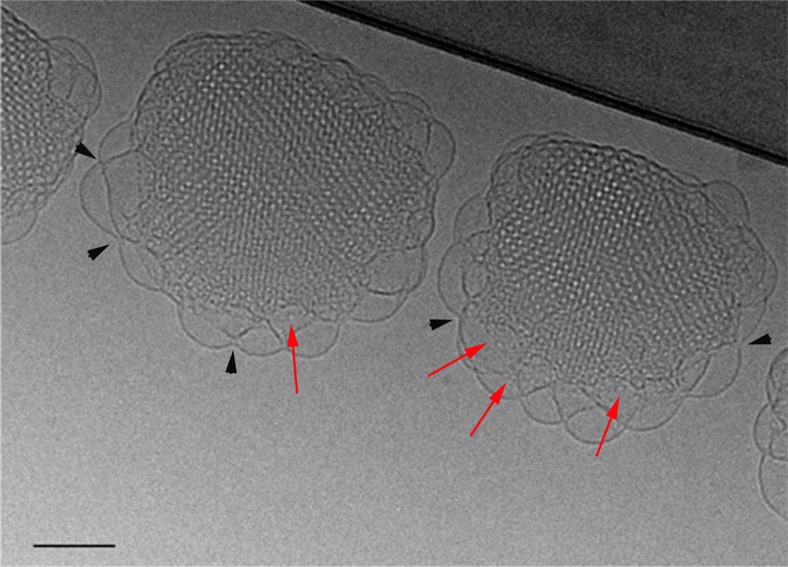
Cubosomes obtained after heating at 100 °C. Note that the resulting particles are surrounded by an extended amount of lamellar structure. Membrane fusion events (short black arrows) and ILAs (long red arrows) are also evidenced. Images were obtained after cubosomes, used in the present work, were heated at 100 °C and cooled down to room temperature. Scale bar, 100 nm.

A further question is whether the stabilization mechanism and the structures described in the present study are at or close to equilibrium. Barauskas *et al*.^37^ used heat treatment at 125 °C to obtain stable dispersed bicontinuous cubic phase at or close to equilibrium. In that case, as shown in supplementary Fig. 12, lamellar phase at the particle/water interface is present and membrane fusion events are evidenced. To confirm that for the composition used in the present work, the stabilization mechanism corresponds to an equilibrium situation, we performed also heating at high temperature and cooling (Fig. 5; Supplementary Fig. 13). Extended amount of lamellar phase surrounds the cubic phase. In addition, with the knowledge gained from the CET analysis (Fig. 4; Supplementary Fig. 7), ILAs and membrane fusion events are easily identified (Fig. 5) in conventional cryo-TEM images. This indicates that the stabilization mechanism proposed here, involving ILAs, corresponds to situations at or close to equilibrium and confirms that its appearance is very general.

In addition, de Campo *et al*.^39^ show that internal structure of the bicontinuous cubic particles was at equilibrium since the crystallographic information obtained from SAXS was identical to the one of the non-dispersed phase and there was no change of structures after heating the dispersion up to 94 °C and cooling back to room temperature. At 94 °C, the particles adopt the reversed microemulsion structure. For the heating experiment performed in the present study, the fast Fourier transform analysis indicates a lattice parameter of the bicontinuous cubic internal structure in the range of 15–16 nm that is in good agreement with the one of the non-heated dispersion (about 16 nm) in accordance with the study of de Campo *et al*.

Our work indicates that the internal structure has essentially no defect, which confirms the study of de Campo *et al*.^39^, which, as mentioned before, shows that the SAXS pattern of a dispersion containing monoglycerides and Pluronic 127 is the same as the one of the bulk phase containing only monoglyceride. This indicates that the particle interior contains little or no stabilizers. Landh^40^ studied the ternary phase diagram Pluronic F127–unsaturated monoglyceride–water. A lamellar phase was observed for compositions containing almost equal amounts of Pluronic 127 and monoglyceride, strongly suggesting that the stabilizing layer is rich in Pluronic. The high content in Pluronic of the disordered vesicular structure external to the particle is also in agreement with the fact that the amount of vesicles increases with the content of Pluronic F127 as mentioned earlier.

From all the above findings and the new experiments carried out here, the structure discussed in the present work is deduced to be at thermodynamic equilibrium.

The cubosome interface reported here is very different from the ones previously reported using electron microscopy imaging. Angelov *et al*.^25^ and Boyd *et al*.^26^ proposed that the particles are composed of repeating units also present at the interface, and no ILAs or disordered vesicular structures at the interface are included. With this model, no composition gradient across cubosomes is expected and only discrete sizes and forms of cubosomes are present, which is not the case for our model. Angelov *et al*. used freeze fracture where the interpretation of the fracture is not always straightforward and where only a cut through the object is visible. Boyd *et al*. used sublimation, which removes water and may affect the particle surface structure. Our work illustrates the power of CET where the full object is imaged in its native state in presence of water, allowing 3D reconstruction at high resolution.

In our model, one water channel is isolated from the outside aqueous matrix by a bilayer as it is the case in Andersson model (Supplementary Fig. 6). If complex vesicular structures are formed at the interface, which represents the majority of the cases, water release from the second channel is restricted to a limited number of locations or is suppressed when a lipid bilayer completely surrounds the cubosome. This situation favours sustained release of solubilized hydrophilic molecules (that is, it decreases the effective release rate). It was recently found that the diffusion coefficient of glucose and proflavine in lamellar phase is between 10 and 100 times lower than the one of this molecule when solubilized in the inverted bicontinuous cubic phase^41^. This also calls for a re-examination of previous models for release of hydrophilic drugs from cubosomes, which assumed direct molecule transfer from inner water channels to external water without bilayer crossing.

The heating and cooling experiments favour the presence of a thick lamellar phase at the particle water interface (Fig. 5; Supplementary Fig. 13) compared with the initial dispersion (Fig. 1; Supplementary Fig. 3). This offers a way to increase the thickness of the lamellar structure. In addition, more stabilizers could be used to achieve this task. Furthermore, in the future, diffusion could be further limited by inserting other molecules (for example, cholesterol) in the vesicular structure. In summary, we use CET to resolve directly the structure of lipid cubosomes. Not only have our results conclusively validated the bicontinuous structure of cubosomes, previously inferred from indirect reciprocal space techniques (X-ray scattering) or molecular transport measurements, such as diffusion, conductivity and self-diffusion NMR, but they also demonstrate that the mechanism of cubosome stabilization involves membrane fusions and ILAs present in the transition between infinite periodic minimal surface and lamellar structures giving insight in the structural variation within the particles. These results advance our understanding of these systems and pave the way to a rational understanding of biological phenomenon associated to reversed bicontinuous cubic phases and molecular transport properties in these fascinating internally structured colloids.

## Methods

### Cubosomes preparation

Pluronic F127 (0.075 g, Sigma) was dispersed into 19 g of milliQ water. Glycerol Monolinoleate (0.69375, g, Emulsifier TSPH039 from Danisco, which has a purity of 93.8% monoglyceride and contains 4.1% diglycerides. The fatty acids are composed of 91.8% linoleate, 6.8% oleate and about 1% saturated fatty acids were mixed with polyglycerol ester 0.23125, g (PGE 080D from Danisco) to form the lipid mixture. The lipid mixture was added to the Pluronic aqueous solution. Ultrasonication was applied at cycle 1 and 70% intensity for 5 min with a probe sonicator (Ultraschallprozessor 400, Hielscher).

### Small-angle X-ray scattering

Laboratory SAXS measurements were performed with a MicroMax-002+ microfocused X-ray machine (Rigaku), operating at 4 kW, 45 kV and 0.88 mA. The Kα X-ray radiation of wavelength *λ*=1.5418 Å emitted at the Cu anode is collimated through three pinholes of respective sizes 0.4, 0.3 and 0.8 mm. The scattered intensity was collected on a two-dimensional Triton-200 X-ray detector (20-cm diameter, 200-μm resolution) for 16 h. The scattering wave vector is defined as q=4*π*sin(*θ*)/*λ*, where 2*θ* is the scattering angle. The sample chamber used gives access to q ranges of 0.01–0.44 Å^−1^. Silver behenate was used for q vector calibration. Scattered intensity data were azimuthally averaged using SAXSgui software (Rigaku). Dispersion samples were filled into 1.5-mm diameter quartz capillaries, sealed with epoxy glue (UHU). The X-ray machine is thermostated at 22.0±0.5 °C, taken as room temperature.

### Cryo-electron microscopy and 3D tomography

A 5-μl sample was deposited onto a lacey copper grid; the excess was blotted with a filter paper, and the grid was plunged into a liquid ethane bath cooled with liquid nitrogen inside a climate chamber (Vitrobot MarkIV, FEI, Eindoven). The climate chamber temperature was 22.5 (±0.5) °C and the relative humidity was kept close to saturation (100%) to prevent evaporation of the sample during preparation. Specimens were maintained at a temperature of −170 °C using a cryo holder 626 (Gatan) and were observed with a JEOL 2200FS electron microscope operating at 200 kV equipped with a omega energy filter and at a nominal magnification of 40,000 under low-dose conditions. Images were recorded with a 2 × 2 k slow scan charge-coupled device camera (Gatan).

For 3D tomography, tilt series were collected automatically from −60° to +60° with 2° angular increment using the Recorder (JEOL) software. Images were recorded on charge-coupled device camera at a defocus level between −2 and −3 μm. For image processing, using colloidal gold particles as fiducial markers, the two-dimensional projection images, binned by a factor of one, were aligned with the IMOD software^42^ and then tomographic reconstructions were calculated by tomoJ^43^.

### Subtomogram averaging

A total of 150 subtomograms of dimension 200 × 200 × 200 pixels (pixel=0.3 nm) were boxed inside the cubosome and next extracted using bsoft^44^. These subtomograms were aligned in 3D using Spider. To compensate the missing wedge and based on the internal symmetry of the cubosome, we then duplicated all extracted boxes and rotated them at 90°. After several iterations of averaging (Supplementary Fig. 14) using the output as a new reference, we obtained a final map and visualized it using ImageJ^45^ and the 3D representation using UCSF Chimera^46^.

### Heating the dispersion at 100 °C

A digital heatblock (VWR International, model number 949307) was set-up at 100 °C. A measure of 10 ml of the dispersion was introduced in Pyrex culture tubes 100 × 26 mm. Once the heating block temperature reached 100 °C, the Pyrex tube was introduced into the heating block and heated for 40 min. After this time, heating was switched off. After 1 h, the dispersion was removed from the heating block.

## Additional information

**How to cite this article:** Demurtas, D. *et al*. Direct visualization of dispersed lipid bicontinuous cubic phases by cryo-electron tomography. *Nat. Commun.* 6:8915 doi: 10.1038/ncomms9915 (2015).

## Supplementary Material

Supplementary Information Supplementary Figures 1-14 and Supplementary References

Supplementary Movie 1 Subtomogram averaging of a cubosome interior. The rendering of the subtomogram averaging confirms the presence of the two alternate continuous water networks highlighted by the yellow slice. The box processed by subtomogram averaging is composed by 27 unit cells equally distributed in the x, y, and z direction. The scanning of the yellow slices shows the evolution of the two water networks and of the lipid network. The observed unit cell has the primitive type symmetry with the space group: Im-3m.

Supplementary Movie 2 Tomogram of isolated vesicular structures. The cubosome dispersion contains also small particles having no crystalline order in their internal core. These consist of intermediate structures characterized by steps of membrane fusion and ILAs formations. Scale bar has a length of 100 nm.

Supplementary Movie 3 Tomogram of a cubosome. Vesicular structures are observed at the interface between the particle and the water matrix. Those are intermediate structures between lamellar liquid crystalline and inverted bicontinuous cubic phases involving membrane fusion and ILAs. These intermediate structures are mainly visible at the beginning and at the end of the video. The inverted bicontinuous cubic phase, without defects, is present in the core of the cubosome. It is visible in the central frames of the video. This tomogram was obtained using gold marker of 15 nm in diameter. The total electron dose applied for the acquisition is of 2400 electrons/nm^2^. The scale bar length is 50 nm.
