# Supplementary material for: Direct visualization of dispersed lipid bicontinuous cubic phases by cryo-electron tomography
Source: Nat Commun. 2015 Nov 17;6:8915. doi: 10.1038/ncomms9915 (PMC4660369; doi:10.1038/ncomms9915)
Supplement: Supplementary Information — Supplementary Figures 1-14 and Supplementary References [file ncomms9915-s1.pdf]

Supplementary Figure 1

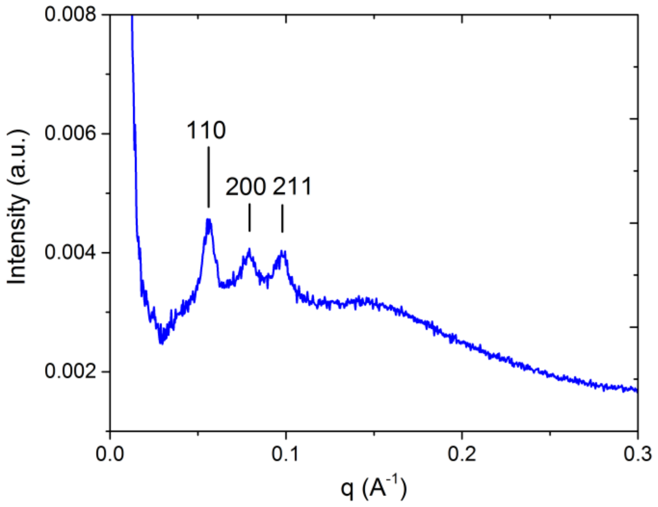

Supplementary Fig.1: SAXS data indicating a Im-3m space group

SAXS measurements at room temperature (22°C) of the cubosome dispersion used in this study correspond to a Im-3m space group symmetry with a lattice parameter of 15.8 nm. This space group determination is done by considering the structures reported for monoglyceride self-assembled structures.[1-4] Those are the inverted bicontinuous cubic structures of symmetry Pn-3m, Ia-3d and Im-3m, the lamellar structure, the hexagonal structure, the micellar cubic structure (space group Fd-3m) and the reversed microemulsion. Composition and processing of this dispersion are indicated in Materials and Methods.

Supplementary Figure 2

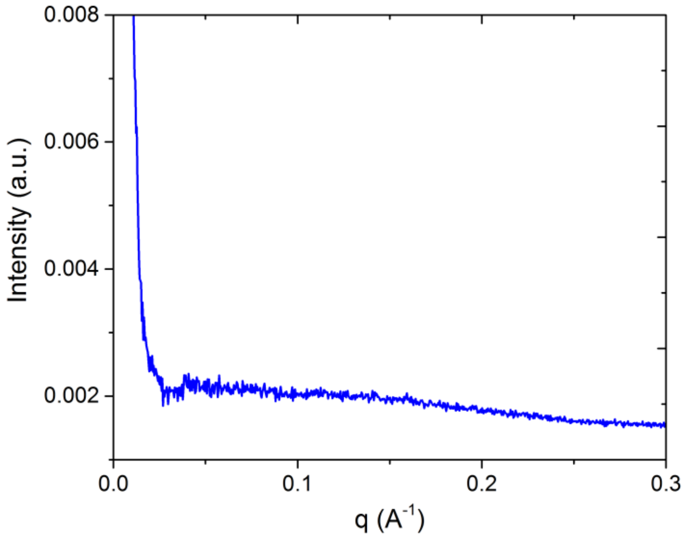

Supplementary Fig. 2: Effect of polyglycerol ester addition on stability of the cubic structure

The cubic structure swells with the addition of polyglycerol ester, resulting in larger water domains that facilitates 3D electron tomography reconstruction. For the present measurement (Supplementary Fig. 2), the ratio polyglycerol ester / glycerol monolinoleate is 33 : 67 instead of 25 : 75 used in Supplementary Fig. 1 and for the rest of the studies. Dispersion composition used in SAXS measurement for Supplementary Fig. 2: 0.075 g Pluronic F127, 19g milliQ water, 0.617 g Glycerol monolinoleate and 0.308 g Polyglycerol ester. The SAXS measurements confirmed that cubic particles were not present in the dispersion solution and had low crystallinity at higher concentrations of polyglycerol ester. Most likely, the dispersion was mainly composed of vesicles. Consequently, the 25 : 75 composition was used for the rest of the study (Methods section) as it results in an increase water domain size while maintaining cubic structure crystallinity.

Supplementary Figure 3

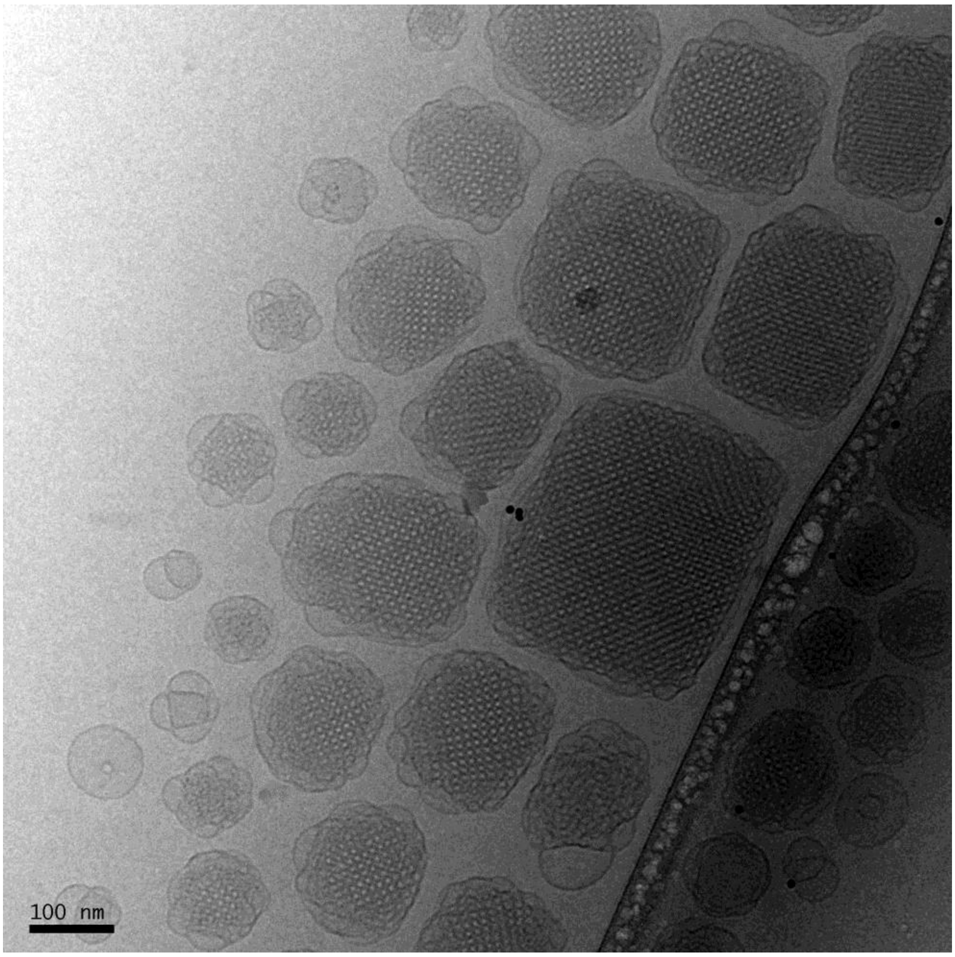

Supplementary Fig. 3: Cryo EM image of the studied dispersion

This image shows well-ordered particles with attached vesicular structures co-existing with the isolated vesicular structures.

Supplementary Figure 4

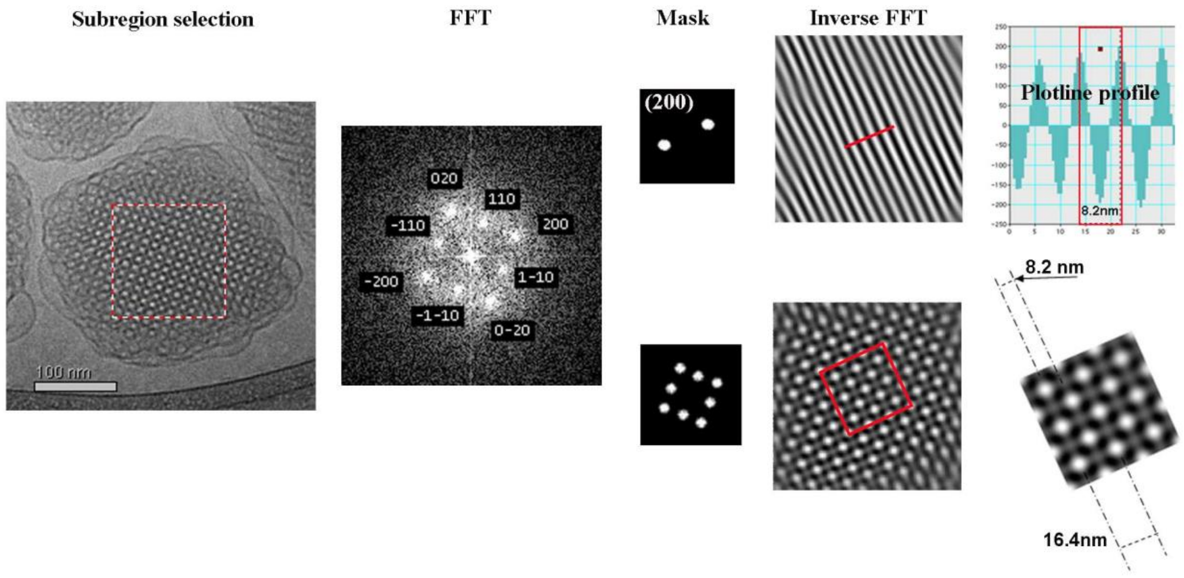

Supplementary Fig. 4: Crystallographic information from Cryo-TEM image

A central region outlined by red square shows a cubosome oriented on its [001] zone axis as determined from the analysis of the spatial frequencies in the adjacent FFT pattern.

To our knowledge in published literature, the only structures reported for monoglyceride self-assembled structures are reverted disordered micellar (L2), lamellar liquid crystalline, columnar hexagonal and cubic[1-4]. Among these structures, the cubic structures are the only ones compatible with a 2D square motif. The Im-3m (200) facets is selectively visualized by masking the corresponding diffraction spots in the FFT pattern. The inverted image is used to measure the distance in the real space of a specific detail on the crystal. The plot profile indicates the distance between the two interdependent networks, which corresponds to half of a unit cell. The hole center to center distance is 16.4 ± 0.4 nm. Considering the fact that SAXS spectra are mainly sensitive to large particle[3], while in Cryo-TEM, only small particles were measured, the agreement between the 16.4 nm measured by cryo-TEM and the 15.8 nm measured by SAXS (Supplementary Fig. 1) is very good.

Supplementary Figure 5

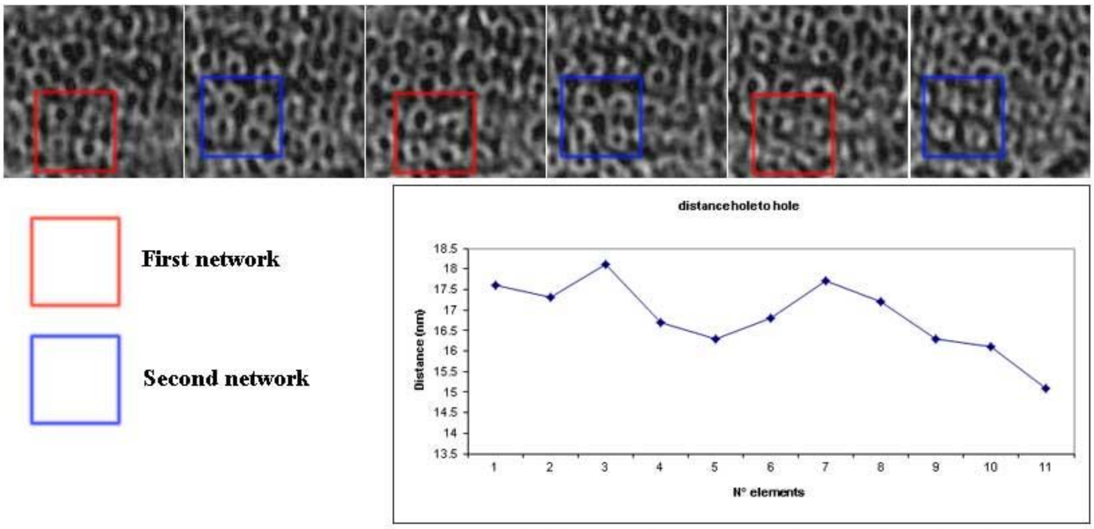

Holes center to center distance = 16.8 nm (± 0.9)

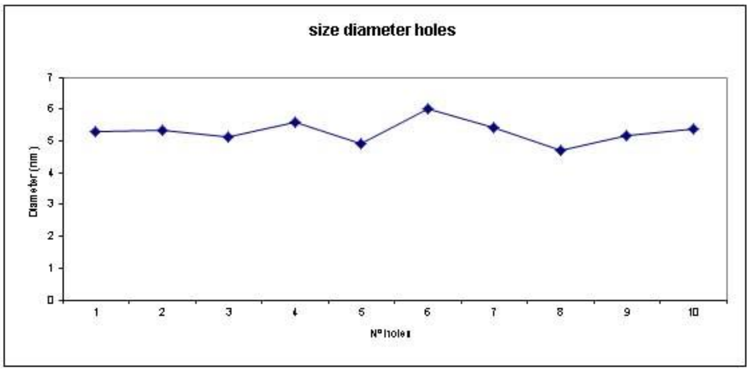

average hole diameter size = 5.3nm (±0.3)

Supplementary Fig. 5 : Determination of the lattice parameter from tomogram slices

From the original tomogram, different z slides were used to measure the distance between water channels. The hole center-to-center distance has been detected manually using the free-software ImageJ (NHI). The data show an average center-to-center distance of 16.8 nm (± 0.9). The fluctuations are due in large part to the resolution of the tomogram. This determination of lattice parameter for an isolated cubosome is a pre-requisite for performing sub-tomogram averaging procedure

Supplementary Figure 6

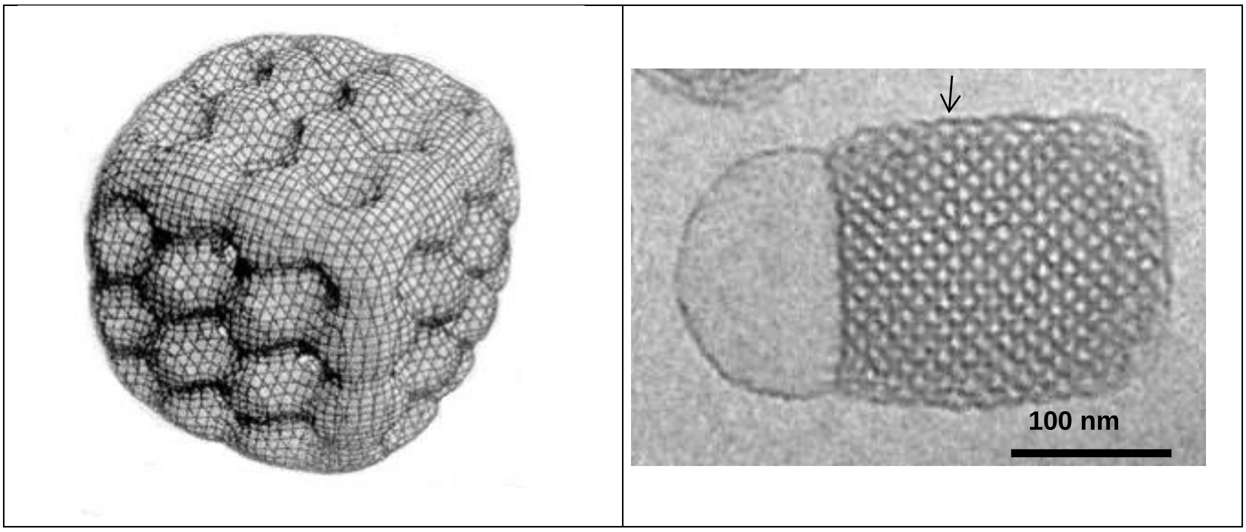

Supplementary Fig. 6: Mechanism of cubosome stabilization proposed by Anderson et al.

Left: Anderson et al.[5] proposed a mechanism for creating stabilized cubosome structures by closing one of the two water channel using a bilayer cap. Though the proposed mechanism is conceptually sound, it does not correspond to the majority of experimental pictures of cubosomes, where disordered vesicular structure are observed at the interface particle/ aqueous matrix[3,4]. Reprinted with permission from Gustafsson et al.[4] Copyright 1997 American Chemical Society.

Right: Cubosomes, where no large disordered vesicular structure is observed on three of these faces. Note that a continuous oscillating membrane (indicated by an arrow) is observed to close the particle. This image matches well the membrane morphology present at the surface of the particle on the left strongly suggesting that for this cubosome (right image), the Andersson mechanism operates.

Picture on the right reprinted with permission of Sagalowicz et al.[3]

Supplementary Figure 7

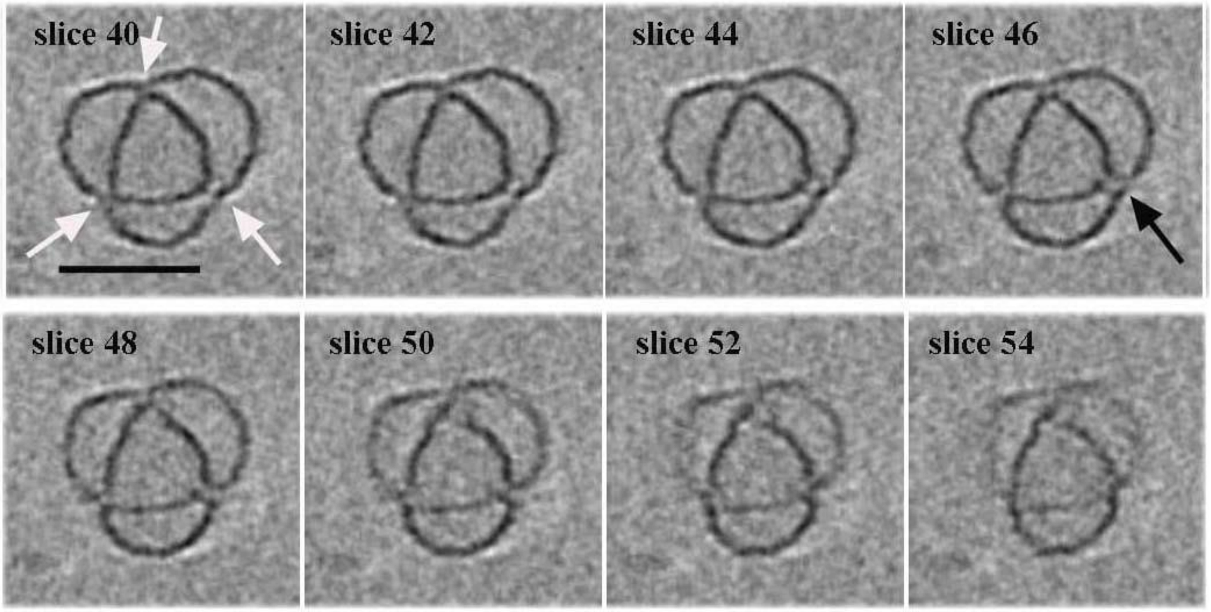

Supplementary Fig. 7: ILAs on side view of isolated vesicular structures

The slices extracted from the vesicle tomogram show the evolution of the membrane in proximity of the ILAs (white arrows). The approach of the two membranes is followed by fusion and ILA formation. The structure, shown here, was an intermediate state between lamellar liquid crystalline and inverted bicontinuous cubic structure that did not transform to cubic structure, as was observed in the case shown Fig. 4. This intermediate state results either because it is too small or has the wrong stoichiometry (e.g. not enough monoglyceride). Slice 46 shows the maximum diameter of the ILA pores (black arrow) visualized in side view for comparison with images shown in Fig. 4. The scale bar is 50nm.

Supplementary Figure 8

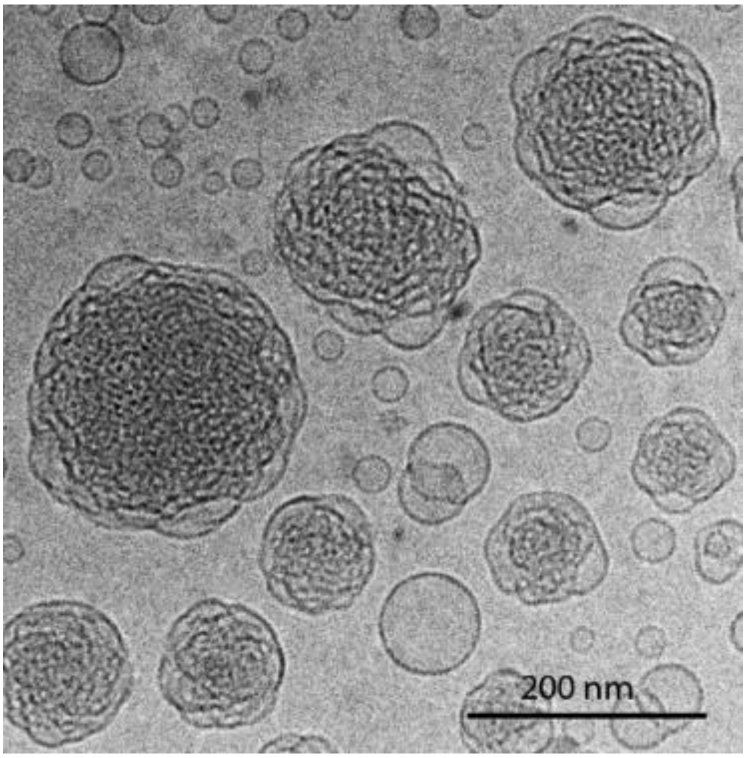

Supplementary Fig. 8: Cubosome stabilized by partially hydrolyzed lecithin

Cryo-TEM image was obtained using a dispersion stabilized by the partially hydrolyzed lecithin (Emultop EP from Cargill; USA). Composition of the dispersion is 4.574% Dimodan U/J, 0.051 wt % vitamin E acetate,0.375 wt % partially hydrolyzed lecithin, and 95 wt % water. SAXS performed on the dispersion indicates the presence of particles having a reversed bicontinuous cubic structure of the primitive type even if some lecithin moves in the particle interior disturbing the structure[6].

Note that the particles display a disordered vesicular structure on the outside as it was the case when using F127 as stabilizer strongly suggesting that the mechanism of stabilization described in the present work, and involving ILAs also operate.

Reprinted with permission from Sagalowicz et al.[6] Copyright 1997 American Chemical Society **.**

Supplementary Figure 9

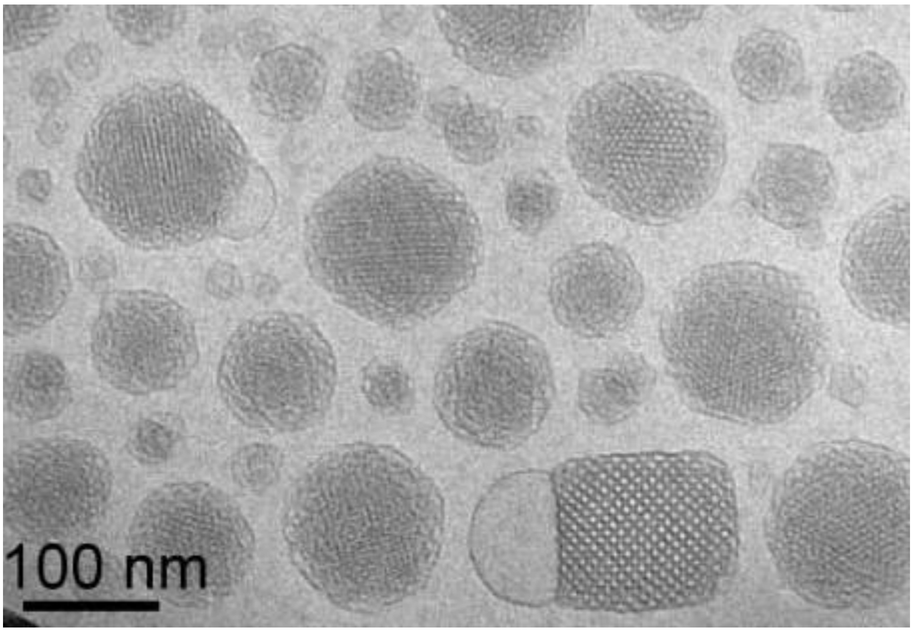

Supplementary Fig. 9: Cubosome stabilized by proteins

Cryo-TEM image was obtained using a dispersion stabilized by proteins (Sodium caseinate). Composition of the solution was 4% monoglyceride (Dimodan U form Danisco), 1% protein and 95% water.

Note that disordered vesicular structures are observed around the particles as it was the case when using ultrasonication strongly suggesting that the mechanism of stabilization described in the present work, and involving ILAs also operate.

The rectangular particle on the bottom part of the image that is oriented along the <100> axis without being surrounded by vesicular structures may be stabilized by the mechanism proposed in Anderson model.[5]

Reprinted with permission of Sagalowicz et al.[3]

Supplementary Figure 10

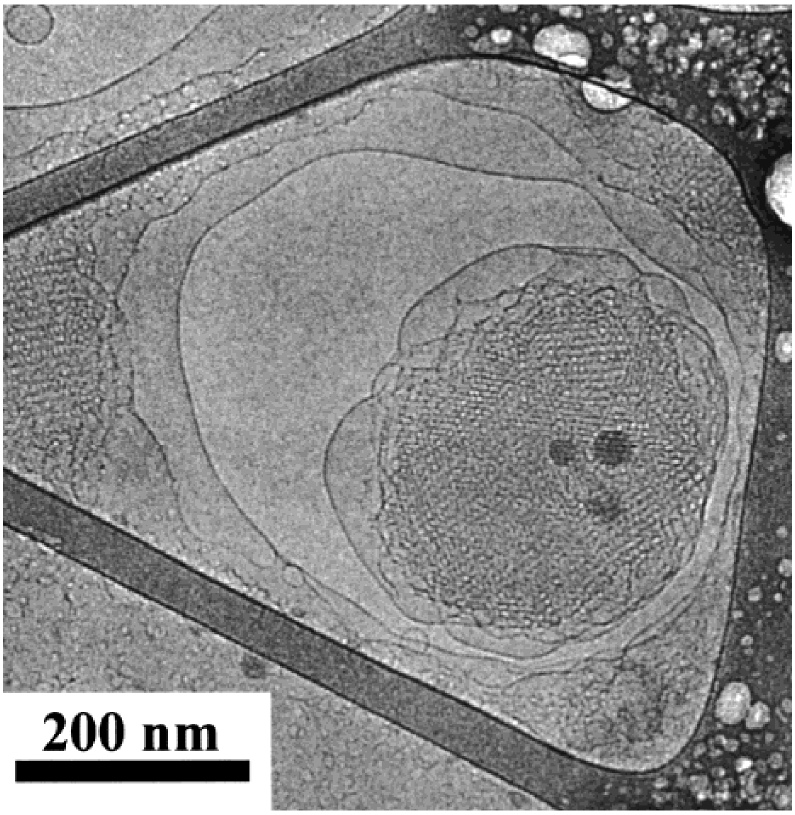

Supplementary Fig. 10: Cubosomes obtained by the hydrotrope route

Cryo-TEM image displaying a cubosome obtained by the hydrotrope route. The hydrotrope route consists in mixing the monoglyceride with ethanol and then adding a Pluronic F127 solution.

Note that a vesicular disordered structure is observed around the particle (similar features are present in other particles obtained by the same process and are discussed further in this reference) as it was the case when using ultrasonication suggesting that the mechanism of stabilization described in the present work, and involving ILAs also operates.

Reprinted with permission from Spicer et al.[7] Copyright 1997 American Chemical Society.

Supplementary Figure 11

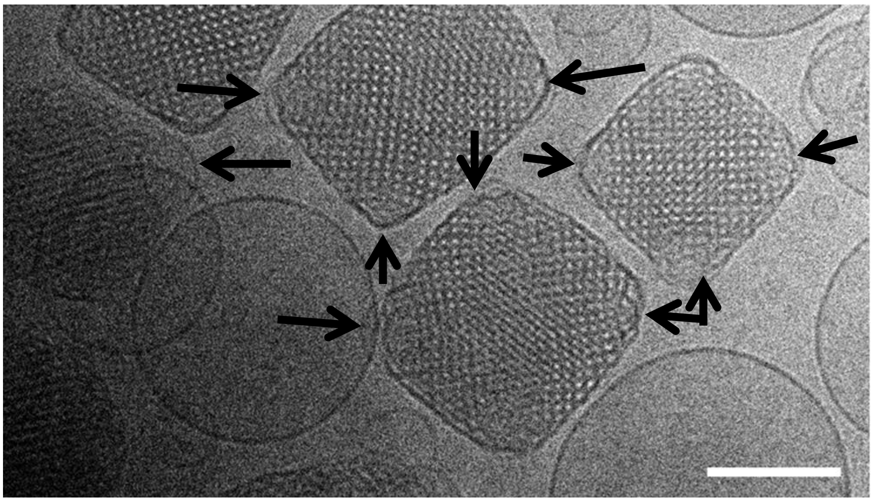

Supplementary Fig. 11: Cubosomes obtained by the evaporation method

Cryo-TEM image displays cubosome structures obtained by the evaporation method. Limonene was added to a mixture containing 10% Dimodan U (Danisco), 0.4% Poloxamer and 89.6% water. Mild ultrasonification was applied, and a dispersed reversed microemulsion (L2) was obtained. The cubic structure forms after complete evaporation and removal of the limonene.

The rectangular particles are oriented normal to the {100} interface planes without the appearance of large vesicular structures around the cubsomes which again suggests a stabilization mechanism according to the Anderson model[5]. However, some disordered vesicular structures are observed in the particle corner, suggesting that the mechanism presented in this work is necessary for stabilization. Scale Bar length is 100 nm.

Reprinted with permission from Martiel et al.[8] Copyright 2014 American Chemical Society.

Supplementary Figure 12

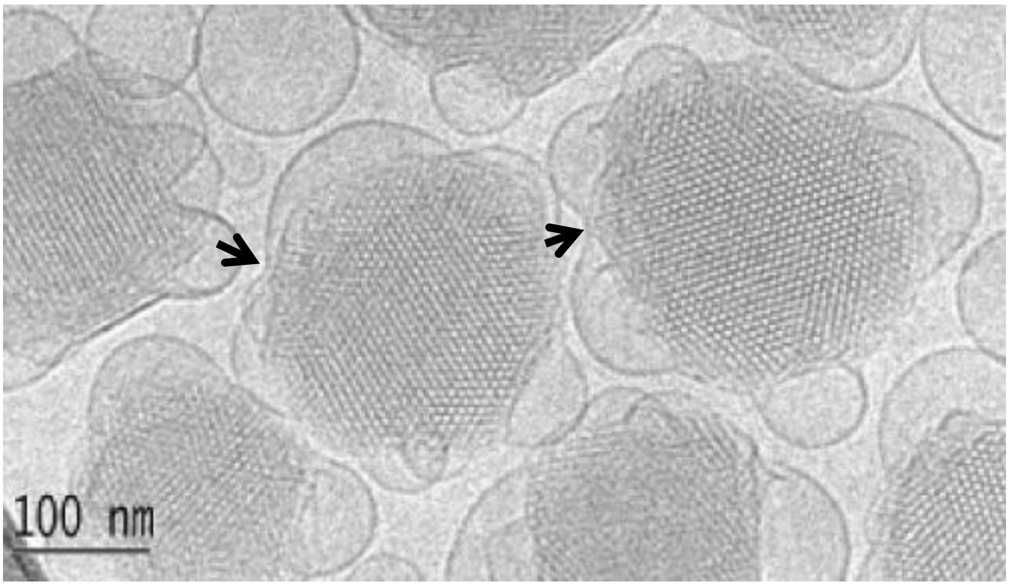

Supplementary Fig. 12: Membrane fusion events observed at the interface cubosome –water for particle having a Pn-3m symmetry

The system is glycerol monooleate (GMO)- oleic acid (OA)-pluronic F127 (ratio GMO:OA, 95:5; ratio lipid: F127, 9:1 and). The symmetry of the space group of the dispersion is Pn-3m.

Note that membrane fusions and ILAs are more difficult to identify with standard cryo-TEM (Supplementary Fig. 12) than tomography. The volume of the object obtained by CET, allows to scan all the z planes of the interface, increasing the possibility to observe events of membrane fusion and ILA formations. This is also the case in standard cryo-TEM images obtained with the dispersion studied in the present work where presence of membrane fusion is difficult to identify with standard cryo-TEM while it is more straightforward with electron tomography. However, by comparing features observed in standard cryo-TEM images with the one observed by electron tomography, it is possible to localize membrane fusion events.

Reprinted with permission from Barauskas et al.[9] Copyright 2005 American Chemical Society.

Supplementary Figure 13

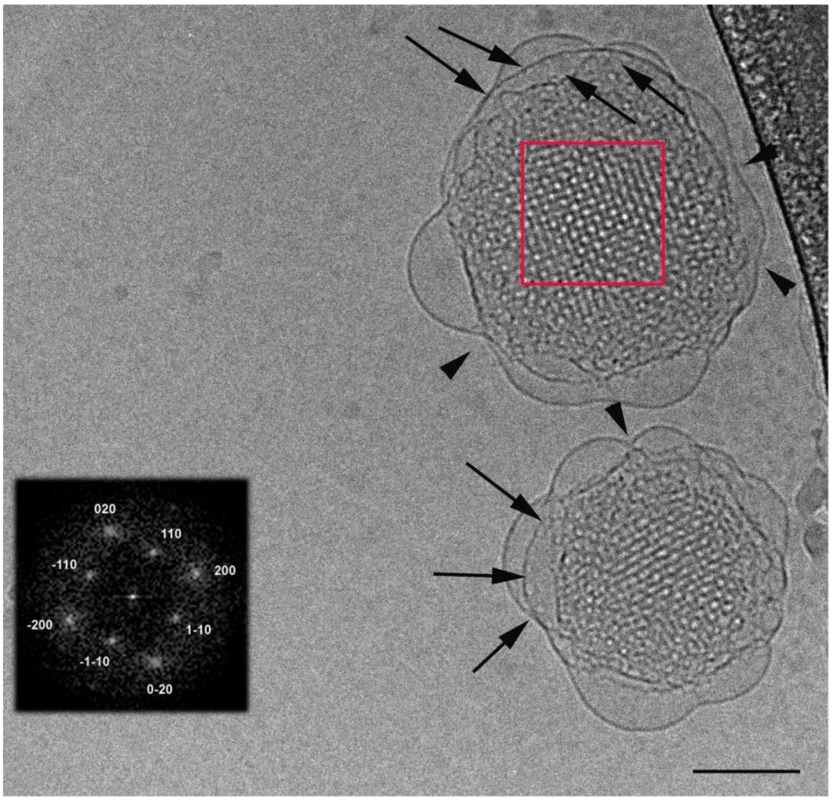

Supplementary Fig. 13: Cryo EM image and FFT (inset) of the studied dispersion after heating at 125°C

Particles with an internal organization are observed. Those are surrounded by lamellar structures. Membrane fusion events (small arrow) and ILAS (large arrows) are also evidenced.

FFT analysis, performed on several particles, leads to an average lattice parameter of 15.6nm in agreement with the lattice parameter of 16 nm determined for the dispersion before heating. Features observed after heating at 125°C are very similar to the ones observed after heating at 100°C.

The particular temperature and heating time were chosen according to the work of Barauskas et al.[9] to obtain a structure at or close to equilibrium.

The heating procedure was performed using a GetinGe GEV 612 autoclave (Sweden) operated at 125°C and 2 bar vapor Pressure. Heating to 125°C took about 9 minutes and it was checked that the dispersion remains for 5 minutes at 125°C controlling the temperature in an identical container filled with water. Scale bar length is 100 nm.

Supplementary Figure 14

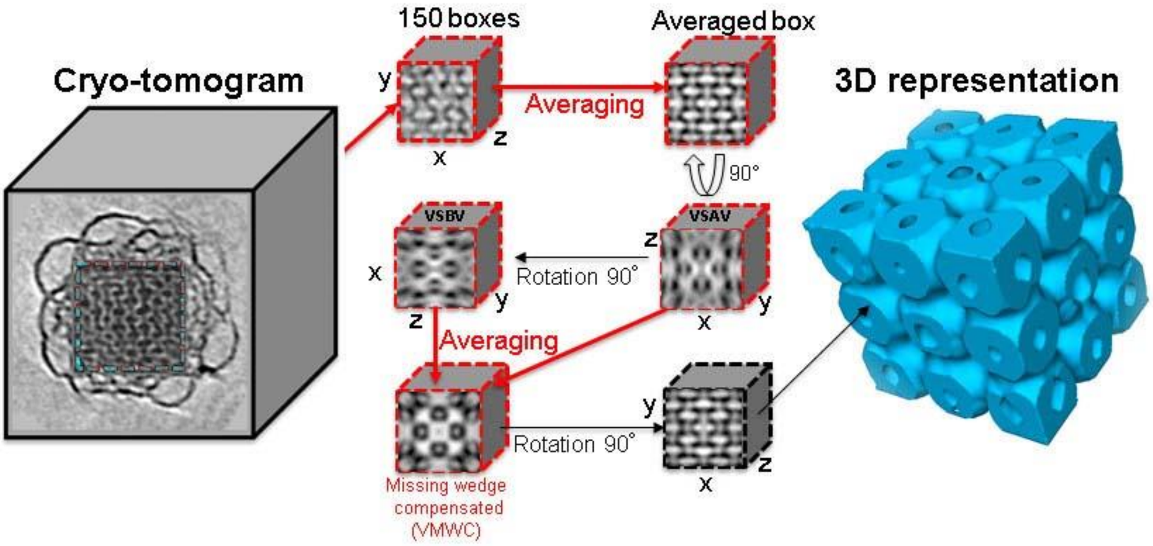

Supplementary Fig. 14: Sub-tomogram averaging

The well-ordered central part of the cubosome was used to produce 150 sub-boxes of 200x200x200 pixels. The boxes differ with each other in their position along the x,y,z directions. The relative displacement between the boxes preserves the periodicity of the cubic centred lattice. The averaging process of the boxes produce a first main volume called "Averaged Box".

To compensate for the missing wedge and using the symmetry of the cubic phase, the averaged box was rotated by 90° along the x axis to produce a new Volume Side-A View (VSAV). An ulterior step rotation along the y axis was used to generate a Volume Side-B View (VBSV). The averaging of VSAV and VSBV produced a volume with the missing wedge compensated (VMWC).

The final 3D reconstruction was obtained by rotating VMWC and it is visualized using UCSF Chimera.

Supplementary references

1 Salentinig, S., Phan, S., Khan, J., Hawley, A. & Boyd, B. J. Formation of Highly Organized Nanostructures during the Digestion of Milk. *ACS Nano* **7**, 10904-10911 (2013).

2 Sagalowicz, L. & Leser, M. E. Delivery systems for liquid food products. *Current Opinion in Colloid & Interface Science* **15**, 61-72 (2010).

3 Sagalowicz, L. *et al.* Crystallography of dispersed liquid crystalline phase studies by cryo-transmission electron microscopy. *Journal of Microscopy* **221**, 110-121 (2006).

4 Gustafsson, M., Ljusberg-Wharen, H., Almgren, M. & Larsson, K. Submicron Particles of reversed lipid phases in water stabilized by a nonionic amphiphilic polymer. *Langmuir* **13**, 6964–6971 (1997).

5 Andersson, S., Jacob, M. & Lidin, S. Structure of the cubosome-a closed lipid bilayer aggregate. *Zeitschrift für Kristallographie* **210**, 315-318 (1995).

6 Sagalowicz, L. *et al.* Influence of vitamin E acetate and other lipids on the phase behavior of mesophases based on unsaturated monoglycerides *Langmuir* **29**, 8222-8232 (2013).

7 Spicer, P. T., & Hayden, K. L., Matthew, L. L., Ofori-Boateng, A. & Burns, J. L. Novel Process for Producing Cubic Liquid Crystalline Nanoparticles (Cubosomes). *Langmuir* **17**, 5748-5756 (2001).

8 Martiel, I., Sagalowicz, L., Handschin, S. & Mezzenga, R. Facile dispersion and control of internal structure in lyotropic liquid crystalline particles by auxiliary solvent evaporation. *Langmuir* **30**, 14452-14459 (2014).

9 Barauskas, J., Johnsson, M., Joabsson, F. & Tiberg, F. Cubic Phase Nanoparticles (Cubosomes): Principles for Controlling Size, Structure and Stability. *Langmuir* **21**, 2569-2577 (2005).
